# Analysis of Influencing Factors in Quantum Chemistry Simulation Based on VQE Algorithm

**DOI:** 10.3390/e28040440

**Published:** 2026-04-13

**Authors:** Meng Zhang, Jian Kang, Qian Wu, Bing Han

**Affiliations:** China National Institute of Standardization, Beijing 100191, China; kangjian@cnis.ac.cn (J.K.); wuqian@cnis.ac.cn (Q.W.); hanb@cnis.ac.cn (B.H.)

**Keywords:** VQE algorithm, quantum chemistry simulation, molecular electronic structure, influencing factors

## Abstract

The Variational Quantum Eigensolver (VQE), as one of the most promising quantum algorithms in the Noisy Intermediate-Scale Quantum (NISQ) era, exhibits unique advantages in quantum chemistry simulations. It provides a novel approach to solving molecular electronic structure problems that are difficult to handle with classical computing. However, the performance of the VQE algorithm in quantum chemistry simulation is jointly affected by multiple factors, and its application in practical scenarios still faces numerous challenges. This paper first outlines the basic principles of the VQE algorithm and its core application scenarios in quantum chemistry simulation. Subsequently, it systematically analyzes the mechanism of the influencing factors, such as molecular system characteristics and algorithm parameter design, focusing on exploring how each factor specifically influences the results. Finally, the current research status and limitations in the optimization of influencing factors are summarized, and future research directions are proposed. This work aims to provide theoretical reference and technical support for improving the performance of quantum chemistry simulation based on the VQE algorithm and promoting its practical application.

## 1. Introduction

The core goal of quantum chemistry simulation [1] is to solve the Schrödinger equation of molecular systems, obtaining key physical and chemical properties such as ground state energy, electron cloud distribution, and chemical bond characteristics. It plays an irreplaceable role in fields such as drug development, material design, and catalytic reaction mechanism research. Classical computing methods (e.g., density functional theory, coupled cluster methods) can achieve high accuracy when dealing with small molecular systems. However, as the molecular size increases and the number of electrons grows, the dimension of the system’s Hilbert space grows exponentially, leading to a sharp rise in computational complexity and making it difficult to achieve both efficiency and accuracy in simulations. This “exponential wall” problem has become an inherent bottleneck in classical quantum chemistry simulations.

Quantum computing, relying on unique quantum properties such as quantum superposition and quantum entanglement, offers the possibility to break through the limitations of classical computing. Among various quantum algorithms, the VQE algorithm, a hybrid quantum–classical algorithm first proposed by Peruzzo et al. in 2014 [2], has become one of the preferred algorithms for quantum chemistry simulation in the NISQ era [3], due to its shallow circuit depth and low requirements for the fault tolerance of quantum hardware. The VQE algorithm prepares a parameterized trial wavefunction (variational ansatz) through a quantum circuit, measures the expected value (energy) of the Hamiltonian corresponding to the trial wavefunction using a quantum computer, and then iteratively optimizes the variational parameters through a classical optimizer. Eventually, it converges to the ground state energy, and the corresponding wavefunction of the molecular system, achieving the core goal of quantum chemistry simulation.

In recent years, scholars worldwide have carried out extensive research on the application of the VQE algorithm in quantum chemistry simulation [4,5,6,7,8,9,10]. Successful simulations of the ground state energy of simple molecular systems, such as hydrogen molecule (H_2_), lithium hydride (L_i_H), and water molecule (H_2_O) systems, have been achieved, and the research has gradually expanded to complex molecular systems. However, in practical applications, the simulation performance of the VQE algorithm is constrained by multiple factors. The interaction among different factors makes it difficult for the computational performance to meet the practical application requirements. For example, noise in quantum hardware leads to errors in energy measurement, unreasonable design of the ansatz makes it difficult for the algorithm to converge to the global optimal solution, and the complexity of the molecular system significantly increases the computational cost.

Recent advances in VQE methodology have significantly expanded its applicability. Tilly et al. (2022) provided comprehensive reviews on VQE methods and best practices, establishing standard benchmarks for chemical accuracy (1 kcal/mol or 1.6 mHa) [11]. Furthermore, recent studies have introduced quantum information-driven ansatz (QIDA) methods that leverage quantum mutual information to construct compact, correlation-driven circuits, showing superior performance in systems ranging from H_2_O to active-space models like N_2_-cc-pVTZ-CAS(6,6) [12]. In terms of ansatz optimization, ADAPT-VQE and its variants (Qubit-ADAPT-VQE) have demonstrated the ability to achieve chemical accuracy with significantly fewer parameters than standard UCCSD-VQE, often by an order of magnitude [13]. Error mitigation techniques, including zero-noise extrapolation and readout error detection, have been shown to improve accuracy by more than 1 mHa compared to unencoded simulations [14]. An advanced ADAPT-VQE scheme with key improvements and a Coupled Exchange Operator (CEO) pool achieves up to 88%, 96%, and 99.6% reductions in CNOT count, depth, and measurement costs for 12–14 qubit molecules, outperforming static UCCSD ansatz across all metrics, with five orders of magnitude lower measurement costs than comparable static alternatives [15]. A folded spectrum VQE extension enables direct excited-state calculation with Pauli grouping for cost reduction and chemical accuracy via error mitigation on noisy simulators [16]. A TETRIS-ADAPT-VQE scheme enabling simultaneous multi-operator addition achieves substantially shallower circuits and reduced gradient measurements, overcoming coherence time constraints on near-term hardware [17]. A differential evolution-optimized VQE scheme achieves superior global convergence for cryptocurrency arbitrage over conventional optimizers, validated on 127-qubit quantum hardware [18]. An ADAPT-VQE scheme with Hamiltonian simplification and optimizer enhancements achieves benzene ground state calculations on IBM hardware, exposing fundamental noise limitations on chemical accuracy [9]. A T-REx error-mitigated VQE scheme achieves an order of magnitude higher ground state energy accuracy on 5-qubit legacy hardware than unmitigated 156-qubit devices, markedly improving variational parameter optimization [19].

At present, most research on the VQE algorithm focuses on algorithm improvement, hardware implementation, or simulation of specific systems. There is still a lack of systematic analysis of the influencing factors of its quantum chemistry simulation, making it difficult to fully reveal the core mechanism affecting VQE simulation performance. Therefore, systematically sorting out the influencing factors of quantum chemistry simulation based on the VQE algorithm is of great theoretical significance and practical value for optimizing the VQE algorithm performance and promoting its practical application in quantum chemistry simulation. Based on the MindSpore Quantum programming toolkit, this paper conducts experimental research on the key factors affecting VQE quantum chemistry simulation from the perspectives of molecular system characteristics and algorithm parameter design, providing a reference for related research.

## 2. Principles of VQE Algorithm and Quantum Chemistry Simulation Process

### 2.1. Basic Principles of VQE Algorithm

The core theoretical basis of the VQE algorithm is the variational principle in quantum mechanics: for any normalized quantum state |ψ(*θ*)⟩ (where *θ* is the vector of variational parameters), the expected value of the corresponding Hamiltonian H, ⟨ψ(*θ*)|H|ψ(*θ*)⟩, is always greater than or equal to the ground state energy E_0_ of the system, i.e., ⟨ψ(*θ*)|H|ψ(*θ*)⟩ ≥ E_0_. The equality holds if and only if |ψ(*θ*)⟩ is the ground state wavefunction |ψ_0_⟩ of the system. Based on this principle, the VQE algorithm gradually approaches the ground state energy and ground state wavefunction of the system through an iterative cycle of “quantum preparation—quantum measurement—classical optimization”.

The variational principle can be expressed in the following form $E_{0}\leq \frac{\langle \psi_{t}\left|\hat{H}\right|\psi_{t}\rangle }{\left\langle \psi_{t}|\psi_{t}\right\rangle }$, where $|\left.\psi_{t}\right\rangle$ denotes the trial wavefunction. The variational principle states that, under certain conditions, the ground state energy obtained from any trial wavefunction is always greater than or equal to the true ground state energy. This principle provides a method for solving the molecular ground state Schrödinger equation: a parameterized function f(θ) is used as an approximation to the exact ground state wavefunction, and the parameters θ are optimized to approach the exact ground state energy.

In the second quantization formalism, the N-electron Hartree–Fock (HF) wavefunction also takes a very concise form: $|\left.\psi_{HF}\right\rangle =\prod_{i=0}^{N-1}a_{i}^{†}|\left.0\right\rangle$. This equation builds a bridge from quantum chemistry wavefunctions to quantum computing: $|\left.0\right\rangle$ represents an unoccupied orbital, and $|\left.1\right\rangle$ represents an occupied orbital. Thus, the *N*-electron HF wavefunction can be mapped to a string of *M* + *N* qubits $|\left.00\ldots 11\ldots \right\rangle$, where *M* is the number of unoccupied orbitals.

We can construct a trial wavefunction of the form $|\left.\psi_{t}\right\rangle =U(\theta )|\left.\psi_{HF}\right\rangle$, where U(θ) denotes a unitary transformation that can be simulated by a quantum circuit, and $|\left.\psi_{HF}\right\rangle$, as the initial state, can be conveniently prepared using multiple single-qubit X gates. The specific form $U(\theta )|\left.\psi_{HF}\right\rangle$ is also referred to as a wavefunction ansatz.

The coupled-cluster theory is a highly efficient wavefunction ansatz, which requires some modifications for implementation on a quantum computer: $|\left.\psi_{UCC}\right\rangle =exp(\hat{T}-{\hat{T}}^{†})|\left.\psi_{HF}\right\rangle$. UCC stands for unitary coupled-cluster theory, where ${\hat{T}}^{†}$ represents the Hermitian conjugate of $\hat{T}$. Thus, $exp(\hat{T}-{\hat{T}}^{†})$ is a unitary operator.

Notably, the unitary coupled-cluster ansatz assumes that the parameters {θ} in the coupled-cluster operator are real. This assumption is valid for molecular systems; however, in periodic systems, the unitary coupled-cluster ansatz can introduce errors due to the neglect of the complex component. This paper will not discuss the application of unitary coupled-cluster theory in periodic systems for the time being.

The core components of the VQE algorithm include three modules: first, the molecular Hamiltonian construction module, which converts the molecular electronic structure problem into a qubit Hamiltonian that can be processed by quantum bits; second, the variational ansatz module, which prepares the trial wavefunction through a parameterized quantum circuit; and third, the hybrid optimization module, which realizes the iterative optimization of variational parameters by combining quantum measurement and classical optimizer. Its core process can be summarized as follows: Firstly, according to the structure of the target molecular system, the corresponding second-quantized Hamiltonian is constructed, and it is converted into a qubit Hamiltonian through fermion-to-qubit mapping, completing the conversion from a classical description to a quantum representation. Secondly, a suitable ansatz circuit is designed to prepare different trial wavefunctions by adjusting the variational parameters θ. Thirdly, a quantum computer is used to measure the expected value of the Hamiltonian corresponding to the trial wavefunction, obtaining the energy estimate under the current parameters. Finally, the energy estimate is input into a classical optimizer, which outputs new variational parameters. The above process is repeated until the energy estimate converges to a stable value. At this time, the energy is the approximate ground state energy of the molecular system, and the corresponding trial wavefunction is the approximate ground state wavefunction.

### 2.2. Core Process of VQE Algorithm in Quantum Chemistry Simulation

Quantum chemistry simulation based on the VQE algorithm needs to combine quantum chemistry theory and quantum computing technology. Its complete process mainly includes four steps, which are closely related; deviations in any step will affect the final simulation results:

- Step 1: Molecular system preprocessing. The structure of the target molecule (such as bond length, bond angle) is clarified, the simulation accuracy requirements are determined, appropriate quantum chemical approximations are selected to simplify the computational complexity of the molecular system, and the electronic configuration of the molecule is determined to lay the foundation for the subsequent Hamiltonian construction.
- Step 2: Molecular Hamiltonian construction and mapping. According to quantum chemistry theory, the second-quantized Hamiltonian of the molecular system is constructed, which mainly includes kinetic energy terms and potential energy terms (electron–electron interaction, electron–nuclear interaction). Then, the fermion operators are converted into qubit operators (linear combinations of Pauli operators) through fermion-to-qubit mapping methods, obtaining the qubit Hamiltonian that can be processed on a quantum computer. This step is the core connecting quantum chemistry and quantum computing, and the choice of mapping method directly affects the complexity and measurement accuracy of the subsequent quantum circuit.
- Step 3: VQE algorithm parameter configuration and quantum simulation. The structure of the ansatz circuit is designed, the number and initial values of variational parameters are determined; a suitable classical optimizer is selected, and parameters such as the number of optimization iterations and convergence threshold are set; quantum hardware (or quantum simulator) is used to run the ansatz circuit, the trial wavefunction is prepared, the expected value of the Hamiltonian is measured, the energy estimate under the current parameters is obtained, and the variational parameters are iteratively optimized through the classical optimizer until the energy converges.
- Step 4: Verification and analysis of simulation results. The ground state energy obtained by the VQE algorithm is compared with the results of classical computing methods (such as coupled cluster method, density functional theory) to verify the simulation accuracy; the error sources in the simulation process are optimized, and the effect of each influencing factor on the simulation results is evaluated. If the accuracy does not meet the requirements, relevant parameters (such as ansatz structure, mapping method, optimizer) need to be adjusted, and the simulation should be performed again.

In summary, the quantum chemistry simulation of the VQE algorithm is a process of synergy among multiple links and parameters. The characteristics of the molecular system, the design of algorithm parameters, the performance of quantum hardware, and the selection of auxiliary technologies all directly affect the accuracy, efficiency, and stability of the simulation. The following sections will systematically analyze these influencing factors.

## 3. Experimental Analysis of Influencing Factors in Quantum Chemistry Simulation Based on VQE Algorithm

The influencing factors of quantum chemistry simulation based on the VQE algorithm include molecular system characteristics, algorithm parameter design, etc. Each type of factor includes multiple specific influencing factors, which are interrelated and synergistic, jointly determining the performance of VQE simulation. The following sections will detail experimental research based on the MindSpore Quantum programming toolkit to explore the impact of various influencing factors on the simulation results and analyze the mechanisms of action of various influencing factors. Considering that the current quantum computer hardware is not yet mature, we use a classical simulator of quantum circuits to implement the VQE algorithm. The classical simulator of quantum circuits is configured with a 4-core CPU, 8 GB RAM, and 256 GB disk.

The following indicators can usually be used to quantify the impacts of various parameters on the performance of VQE algorithm.

(1) Circuit Complexity Indicators
  - Number of quantum gates *N*_gate_: Total count of single-qubit gates and two-qubit gates.
  - Circuit depth *D*: Number of gate operation layers along the critical path.
  - Number of parameters *N*_param_: Total count of the variational parameters *θ*_i_.
(2) Information-Theoretic Complexity Indicators
  - Quantum mutual information $I_{A,B}=\left(S\left(\rho_{A}\right)+S\left(\rho_{B}\right)-S\left(\rho_{A,B}\right)\right)\left(1-\delta_{A,B}\right)$, where $S\left(\rho \right)=-Tr(\rho log\left(\rho \right)$ is the von Neumann entropy. This indicator quantifies the degree of entanglement between qubits. A higher mutual information indicates that a deeper circuit is required to accurately represent the ground state wavefunction.
  - KL divergence $KL\left(P_{VQE}||P_{FCI}\right)=\sum_{i}P_{VQE}\left(i\right)log\frac{P_{VQE}\left(i\right)}{P_{FCI}\left(i\right)}$, which measures the difference in occupation number distributions between the VQE solution and the exact Full Configuration Interaction (FCI) solution.
(3) Computational Resource Indicators
  - Number of optimization iterations *N*_iter._
  - Number of measurements per iteration *N*_shot._
  - Total computation time *T*_total_ = *N*_iter_ × *T_s_*_ingle_.

### 3.1. Molecular System Complexity

The complexity of a molecular system is mainly determined by the molecular size (number of atoms, number of electrons) and chemical bond types (single bond, double bond, triple bond, conjugated bond). Its impact on VQE simulation is mainly reflected in two aspects: First is the complexity of the Hamiltonian. The larger the molecular size and the more electrons, the more terms in the second-quantized Hamiltonian. The number of Pauli terms in the mapped qubit Hamiltonian increases significantly, leading to a significant increase in the complexity of quantum circuit preparation and measurement, and an increase in the statistical error of energy measurement. Second is the difficulty in designing the ansatz circuit. The ground state wavefunction of a complex molecular system has stronger quantum entanglement characteristics, requiring a more complex ansatz circuit to accurately approximate the ground state wavefunction. If the expressive ability of the ansatz circuit is insufficient, the algorithm will be difficult to converge to the global optimal solution, and the simulation accuracy will be greatly reduced.

We selected several simple molecular structures, such as H_2_, H_4_, H_6_, and H_8_, for simulation experiments. The minimal basis set (STO-3G) was chosen for the basis set, the bond length was fixed at 1.5 Å, and the BFGS algorithm was selected as the classical optimizer.

**Figure 1:**
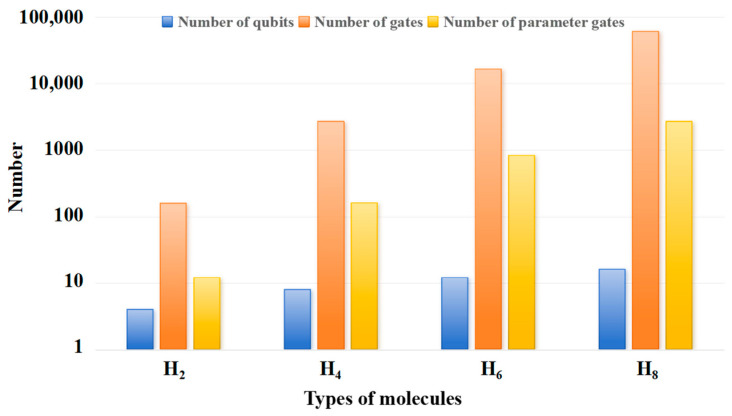
The influence of molecular complexity on quantum circuit complexity.

Figure 1 shows the influence of molecular complexity on quantum circuit complexity. The more complex the molecule, the greater the number of quantum bits and quantum logic gates required, which, in turn, requires more computational resources and higher requirements for the hardware performing the computation.

**Figure 2:**
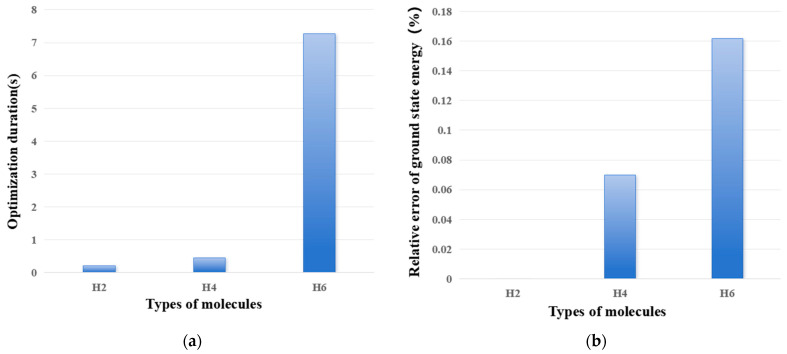
The influence of molecular complexity on optimization time (**a**) and relative error of ground state energy. (**a**) Optimization duration; and (**b**) relative error of ground state energy.

Figure 2 shows the influence of molecular complexity on optimization time and relative error of ground state energy. Here, the relative error refers to the relative error between the ground state energy, calculated by the VQE algorithm, and the exact solution of the ground state energy, calculated by Full Configuration Interaction (FCI). Figure 2a shows that, as the molecular complexity increases, the optimization time presents a rapid growth trend. When calculating the H_8_ molecule, computational resources were exhausted, preventing convergence from being achieved. Figure 2b shows that, as the molecular complexity increases, the accuracy of the VQE algorithm decreases.

### 3.2. Selection of Basis Set

The basis set is the foundation for describing the electronic wavefunction in quantum chemistry simulation. Its selection directly affects the construction accuracy and computational complexity of the Hamiltonian, thereby affecting the simulation performance of the VQE algorithm. There are various types of basis sets, which can be divided into minimal basis sets (such as STO-3G), split-valence basis sets (such as 6–31 G), polarized basis sets (such as cc-pVDZ), and diffuse basis sets (such as aug-cc-pVDZ) according to accuracy: minimal basis sets have the lowest computational complexity but poor accuracy, and are suitable for preliminary simulation of simple molecular systems; split-valence basis sets, polarized basis sets, and diffuse basis sets have gradually improved accuracy, but the size of the basis set gradually increases, leading to a significant increase in the number of terms in the Hamiltonian, and the complexity and measurement error of the quantum circuit also increase accordingly. The following section selects different basis sets to calculate the ground state energy of the H_2_ molecule.

**Figure 3:**
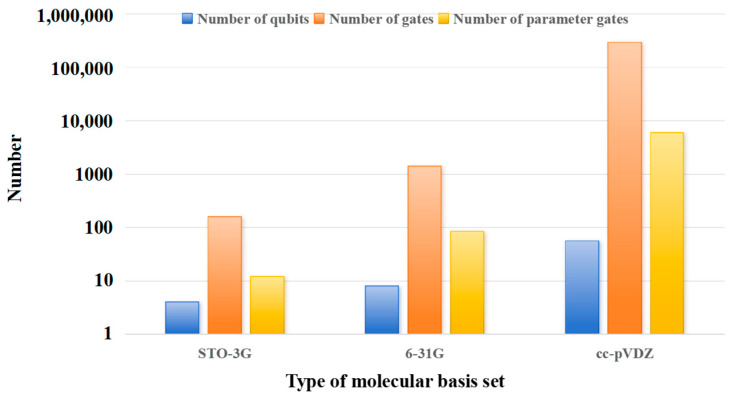
The influence of basis set on quantum circuit complexity.

Figure 3 shows the influence of basis set on quantum circuit complexity. With the increase in the complexity of the basis set, the numbers of quantum bits and quantum logic gates required increase significantly. Since the H_2_ molecule is very simple, the accuracy of the obtained ground state energy is very high, regardless of whether the basis set is STO-3 G or 6–31 G. However, the optimization time of 6–31 G is nearly twice that of STO-3G. When calculating the cc-pVDZ basis set, due to the excessive computational load, timeout and process crash occur, making it impossible to obtain the calculation result. Therefore, in VQE quantum chemistry simulation, it is necessary to select a basis set that balances accuracy and complexity according to the simulation accuracy requirements and the performance of the processor hardware, avoiding insufficient accuracy due to an overly simple basis set, or difficulty in computation due to an overly complex basis set.

### 3.3. Bond Length

Changes in bond length affect the degree of atomic orbital overlap, as well as the molecular orbital layout and energy levels, thereby changing the ground state energy of the molecule. We selected the L_i_H molecule, STO-3G basis set, and modified the bond length to explore its influence on the VQE algorithm.

Bond length variation does not affect circuit complexity (qubit number and Pauli terms remain constant under fixed basis set); hence, circuit metrics are identical across all bond lengths.

**Figure 4:**
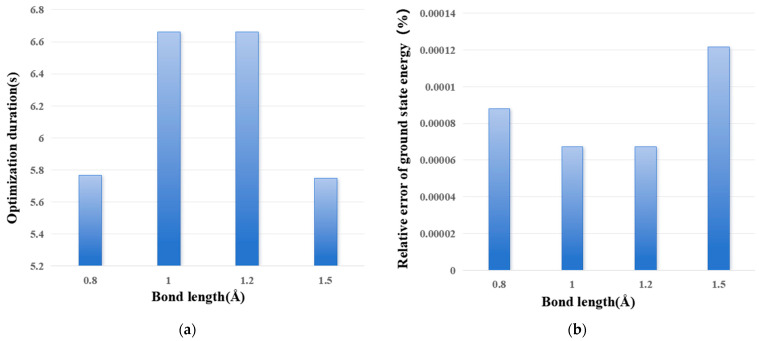
The influence of bond length on optimization time (**a**) and relative error of ground state energy. (**a**) Optimization duration; and (**b**) relative error of ground state energy.

Figure 4 shows the influence of bond length on optimization time and relative error of ground state energy. It can be seen that bond length has no obvious influence on either.

### 3.4. Selection of Optimizer

As a hybrid quantum–classical algorithm, the performance of the classical optimizer directly determines the optimization efficiency and convergence effect of variational parameters, thereby affecting the simulation accuracy and efficiency. The optimization goal of the VQE algorithm is to minimize the expected value of the molecular Hamiltonian (energy). This is a nonlinear unconstrained optimization problem. The optimization difficulty mainly depends on the number of ansatz circuit parameters, as well as the smoothness and non-convexity of the energy function: the more parameters there are, the stronger the non-convexity of the energy function, and, thus, the greater the optimization difficulty.

We selected the LiH molecule, STO-3G basis set, and a bond length of 1.5 Å, and explored the influences of different types of optimizers on the VQE algorithm by modifying the type of optimizer.

In this experiment, we selected five classic optimizers: BFGS, L-BFGS-B, CG, COBYLA, and COBYQA. The CG algorithm iteratively updates parameters by constructing conjugate directions, and its iteration efficiency is higher than that of the traditional gradient descent method; BFGS and L-BFGS-B, as quasi-Newton methods, do not need to calculate the exact Hessian matrix, and approximately update the inverse of the Hessian matrix through gradient information, balancing convergence speed and computational complexity. In addition, L-BFGS-B can handle parameter boundary constraint problems, adapting to some constrained VQE parameter optimization scenarios. COBYLA is suitable for VQE parameter optimization with inequality constraints, and COBYQA is suitable for unconstrained or equality-constrained scenarios. Both can realize parameter iterative update without gradient information.

**Figure 5:**
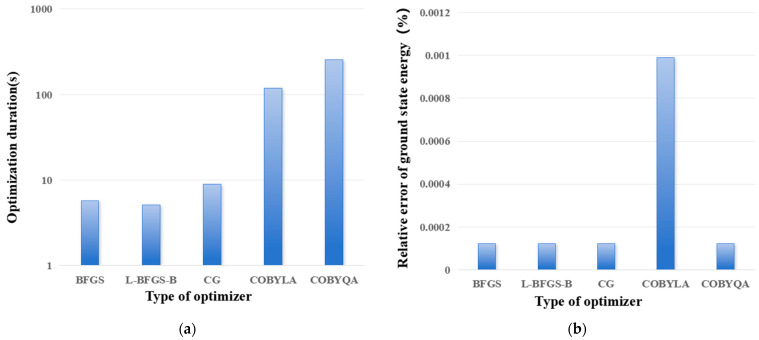
The influence of type of optimizer on optimization time (**a**) and relative error of ground state energy. (**a**) Optimization duration; and (**b**) relative error of ground state energy.

Figure 5 shows the influence of optimizer type on optimization time and relative error of ground state energy. It can be seen that gradient-free optimizers require much longer optimization time than gradient-based optimizers. There is no significant difference in the computational accuracy (relative error of ground state energy) among the four optimizers, BFGS, L-BFGS-B, CG, and COBYQA, while the result of the COBYLA optimizer is significantly inferior to the other four optimizers.

COBYQA outperforms the COBYLA optimizer in computational accuracy for the following reasons: Firstly, it optimizes the approximation strategy, fitting the non-convex energy function of VQE more accurately with a smaller accuracy drop in medium-scale parameter optimization. Secondly, its superior iterative step size adjustment mechanism enables more stable convergence. Thirdly, it adapts to multiple scenarios like unconstrained and equality-constrained optimization, breaking COBYLA’s scenario and scale limitations. Fourth, its improved search strategy boosts global exploration for non-convex functions, avoiding local optima. However, the cost is that COBYQA requires a longer optimization time.

It should be noted that this result comparison is based on simple small molecules, such as L_i_H. For complex molecular structures, the selection of optimizers needs to be discussed in a targeted manner. A balance should be sought among various factors, such as computational efficiency and computational accuracy, according to the requirements of specific application problems.

### 3.5. Comprehensive Benchmarking

**Figure 6:**
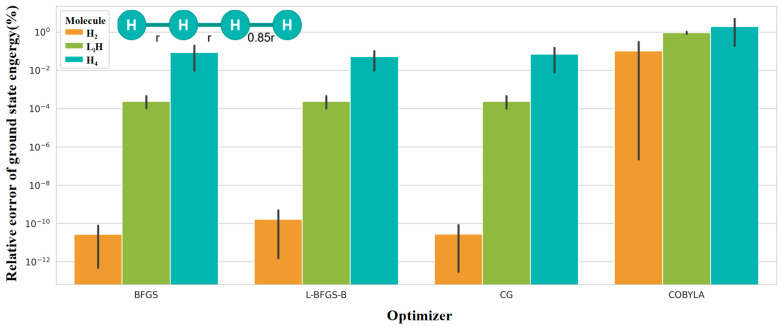
Relative error versus optimizer and molecular system.

In this work, we systematically benchmarked the variational quantum eigensolver (VQE) with the unitary coupled-cluster singles and doubles (UCCSD) ansatz across small molecular systems (symmetric molecules, like H_2_; asymmetric molecules, like LiH; and symmetric molecules with asymmetric bond lengths, like H_4_) under the minimal STO-3G basis set, using four classical optimizers (BFGS, L-BFGS-B, CG, and COBYLA) over a range of internuclear distances. H_4_ with asymmetric bond lengths is shown in Figure 6. The quantum circuit simulations were performed on a statevector simulator, where the Hartree–Fock reference state was prepared, followed by the UCCSD variational unitary. Considering the larger computational complexity of the comprehensive benchmark, in this part of the experiment, the classical simulator of quantum circuits was configured with a 64-core CPU, 128 GB RAM, and 256 GB disk.

Figure 6 illustrates the relative error of VQE energy with respect to the FCI exact solution for H_2_, LiH, and H_4_ systems under the STO-3G basis set, using four optimizers (BFGS, L-BFGS-B, CG, and COBYLA). The abscissa represents the optimizer type, the ordinate denotes the relative error (in percentage), and different colors distinguish molecular systems. The results intuitively reflect the precision discrepancy between gradient-based and gradient-free optimizers, reveal the influence of molecular complexity on VQE solution accuracy, and demonstrate the increased optimization difficulty arising from enhanced electron correlation.

**Figure 7:**
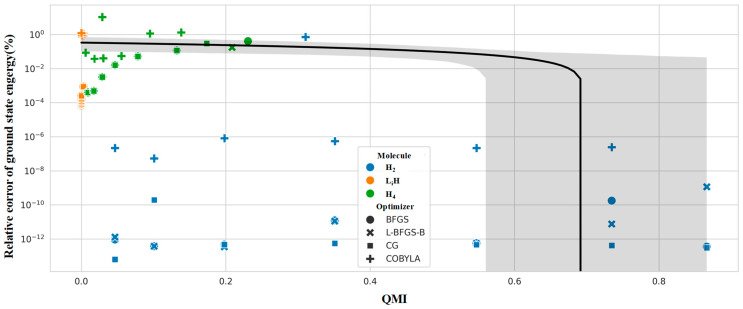
Correlation between quantum mutual information and VQE relative error.

Figure 7 exhibits the relationship between quantum mutual information (QMI) and relative energy error, with an overlaid linear regression fitting curve. The abscissa is quantum mutual information, and the ordinate is the relative error. This figure establishes the quantitative correlation between the strength of many-body correlation and VQE precision.

**Figure 8:**
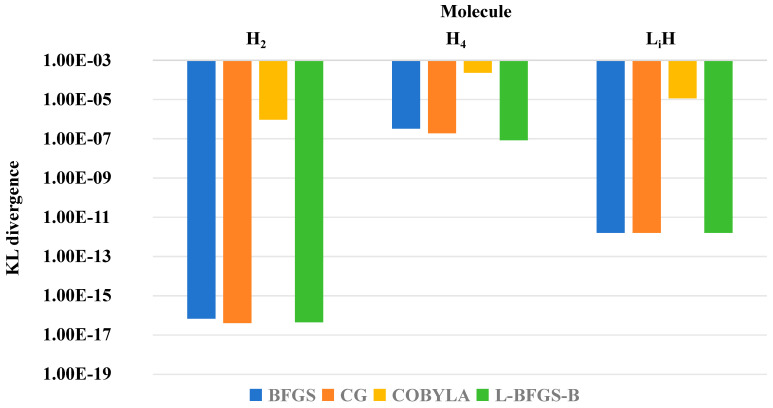
Distribution of KL divergence across molecular systems and optimizers.

Figure 8 presents the statistical results of KL divergence for different molecules and optimizers. The abscissa represents molecular systems, the ordinate denotes KL divergence, and different colors distinguish optimizers. KL divergence quantifies the distribution difference between the VQE approximate quantum state and the FCI exact quantum state, with smaller values indicating higher quantum state fidelity. This figure characterizes solution accuracy at the quantum state level and reflects the deviation of state approximation induced by increased molecular complexity.

**Figure 9:**
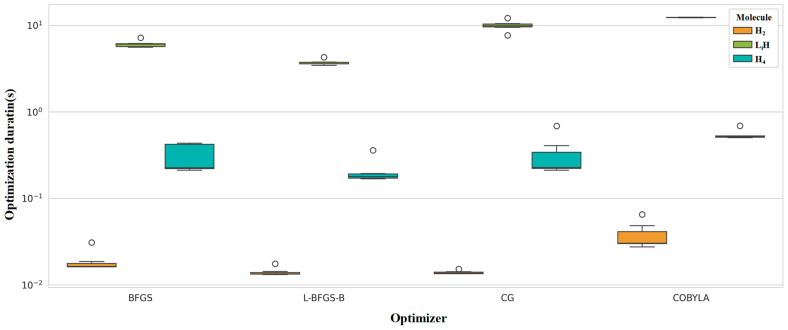
Distribution of total computation time across optimizers and molecular systems.

Figure 9 shows the total computation time for different molecules and optimizers. The abscissa is the optimizer type, the ordinate represents computation time, and different colors distinguish molecular systems. From an engineering efficiency perspective, this figure compares the performance difference between gradient-based and gradient-free optimizers, illustrates the impacts of quantum bit count, number of variational parameters, and circuit scale on computation overhead, and provides data support for the efficiency–accuracy trade-off in practical simulations.

Accuracy dimension analysis reveals that solver precision in the variational quantum eigensolver (VQE) is jointly governed by optimizer type and molecular complexity. Gradient-based optimizers, including BFGS, L-BFGS-B, and CG, achieve efficient search via gradient guidance. Their relative errors are generally maintained below 1%, with L-BFGS-B delivering the best performance. In contrast, the gradient-free optimizer COBYLA yields significantly larger errors, due to its reliance on random search.

At the molecular level, relative errors follow the increasing order H_2_ < LiH < H_4_. This trend arises because many-electron systems exhibit stronger electron correlation and more complex nonlocal interelectronic interactions. The expressive power of the unitary coupled-cluster singles and doubles (UCCSD) ansatz gradually approaches its limit. As a result, the accuracy of both energy approximation and quantum state fidelity decrease synchronously.

Quantitative analysis using quantum mutual information (QMI) uncovers a fundamental link between quantum correlation and solution accuracy. As a quantum-informatic descriptor of electron correlation strength, QMI shows a strong negative correlation with relative error. Physically, this trend reflects the enhanced non-independence of electron motion in strongly correlated systems. The conventional UCCSD ansatz fails to accurately describe many-body correlation effects, increasing the deviation between the prepared quantum state and the exact full configuration interaction (FCI) state.

Bond-length-dependent behavior further validates this mechanism. Near the equilibrium bond length (e.g., 0.75 Å for H_2_), the molecular structure is stable, and electron correlation remains moderate. VQE readily converges to high-precision solutions with the lowest Kullback–Leibler (KL) divergence. When bond lengths deviate from equilibrium, the potential energy surface becomes more steeply curved, and electron cloud overlap is distorted—either excessively overlapping at overly short bond lengths or weakly overlapping at overly long distances. Correlation strength is, thus, significantly disrupted, requiring 1~2 times more optimizer iterations to reach the minimum than at equilibrium.

Analysis of computational efficiency and resource overhead shows that gradient-based optimizers owe their speed advantage to precise exploitation of potential energy surface topology. Linear increases in quantum circuit complexity substantially raise hardware overhead for quantum state evolution and gradient evaluation.

These results demonstrate that the performance limit of VQE simulations for small- and medium-sized molecules is determined by synergy among three factors: optimizer search efficiency, ansatz expressive power, and molecular correlation strength. L-BFGS-B is the optimal optimizer under the STO-3G basis set. High-precision simulations of H_2_ and LiH are feasible on existing noisy intermediate-scale quantum (NISQ) devices. However, strongly correlated systems, such as H_4_, require advanced strategies to overcome accuracy bottlenecks, including higher-order ansatz or circuit optimization. As a key predictive metric, quantum mutual information effectively guides the matching of target systems and algorithm parameters. It provides a quantitative foundation for practical applications of quantum chemistry simulations in the NISQ era.

## 4. Conclusions and Outlook

### 4.1. Conclusions

This paper focuses on quantum chemistry simulations based on the VQE algorithm, systematically analyzes the core factors affecting its accuracy and efficiency, and draws the following key conclusions:

Firstly, the characteristics of the molecular system are the foundation of VQE simulation. Factors such as molecular system complexity and the selection of basis sets determine the computational complexity and the upper limit of simulation accuracy. Complex molecular systems and high-precision basis sets increase the complexity of the Hamiltonian and the difficulty of ansatz design, which reduces simulation efficiency, but can improve simulation accuracy. A balance between accuracy and complexity needs to be sought.

Secondly, the design of algorithm parameters is the core of VQE simulation. Factors such as ansatz circuit design and classical optimizer selection directly affect the convergence of the algorithm and the simulation performance. An ansatz circuit that balances expressive ability and simplicity, and a classical optimizer suitable for the scenario are the keys to achieving high-precision and high-efficiency simulation. Different parameters need to be synergistically optimized.

In addition, the above influencing factors are not independent of each other, but have significant synergistic effects, especially for complex molecular systems.

### 4.2. Future Outlook

Combined with the current research status and limitations, the future research on the influencing factors and optimization strategies of quantum chemistry simulation based on the VQE algorithm can focus on the following three directions:

Firstly, conduct in-depth research on multi-factor synergistic optimization, establish a multi-factor coupling model, reveal the mutual influence law among various factors, propose a global synergistic optimization strategy, and combine technologies, such as machine learning and reinforcement learning, to realize the synergistic adaptation of molecular system characteristics, algorithm parameters, and quantum hardware performance, breaking through the limitations of single-factor optimization and achieving the overall improvement of VQE simulation performance.

Secondly, strengthen the adaptability research on complex molecular systems. According to the characteristics of medium and large molecular systems, and strongly correlated systems, design a new type of ansatz circuit with high efficiency and high expressive ability, optimize the Hamiltonian simplification strategy and mapping method, promote the application of the VQE algorithm in practical scenarios, such as drug development and material design, and enhance the practical value of VQE simulation.

Thirdly, expand the application scenarios of the VQE algorithm. Combined with specific application requirements (such as catalytic reaction mechanism, biomolecular simulation), analyze the influencing factors in a targeted manner, propose personalized optimization strategies, establish a VQE simulation performance evaluation system, and provide theoretical reference and technical support for factor optimization in practical applications; at the same time, strengthen interdisciplinary cooperation, integrate technologies from multiple disciplines, such as quantum chemistry, quantum computing, and computer science, and promote the industrial application of the VQE algorithm.

In the future, with the improvement of quantum hardware performance, the optimization of algorithm design, and the maturity of auxiliary technologies, quantum chemistry simulation based on the VQE algorithm will gradually break through the existing bottlenecks, play a more important role in solving molecular electronic structure problems that are difficult to handle with classical computing, and provide a new technical path for the development of fields such as drug development, material design, and catalytic science.

## Data Availability

Data is contained within the article.
